# Hyperbolic Cross Truncations for Stochastic Fourier Cosine Series

**DOI:** 10.1155/2014/265031

**Published:** 2014-07-24

**Authors:** Zhihua Zhang

**Affiliations:** College of Global Change and Earth System Science, Beijing Normal University, Beijing 100875, China

## Abstract

Based on our decomposition of stochastic processes and our asymptotic representations of Fourier cosine coefficients, we deduce an asymptotic formula of approximation errors of hyperbolic cross truncations for bivariate stochastic Fourier cosine series. Moreover we propose a kind of Fourier cosine expansions with polynomials factors such that the corresponding Fourier cosine coefficients decay very fast. Although our research is in the setting of stochastic processes, our results are also new for deterministic functions.

## 1. Introduction 

For approximations of multivariate functions by algebraic/trigonometric polynomials on full grids, the approximation rate deteriorates rapidly as the dimension *d* increases [1, 2]; this is just so-called “dimension curse” problem. In order to solve it, hyperbolic cross approximations have received much attention in recent years [1, 3–5]. For multivariate periodic functions, Griebel and Hamaekers [4] discussed hyperbolic cross trigonometric approximation and gave the corresponding error estimate. Instead of trigonometric polynomial space on full grids, they used the hyperbolic cross space:

(1) $$\begin{array}{c} X_{N}=\mathrm{span}\left\{\exp\left(ik\cdot x\right):{\left|k\right|}_{\text{mix}}=\overset{d}{\underset{j=1}{\prod }}\max\left\{\left|k_{j}\right|,1\right\}\leq N\right\}, \end{array}$$

as approximation space. In 2010, Shen and Wang [1] studied the hyperbolic cross Jacobi polynomial approximation for functions on the unit cube and gave various formulas on error estimates. Moreover, they also considered the hyperbolic cross Hermite/Laguerre polynomial approximation. In 2009, Boyd [6] deeply researched large-degree asymptotics for Fourier, Chebyshev, and Hermite coefficients of analytic functions. For high-dimensional polynomial interpolation approximation, one often uses tensor product method to generalize one-dimensional interpolation polynomial approximation with Chebyshev knots. In 2000, Barthelmann et al. [3] showed that when the dimension is larger than 10, it is suggested to use sparse grids instead of full grids. Moreover, Barthelmann et al. obtained the corresponding error estimates.

Up to now, for various hyperbolic cross approximation, the asymptotic formulas of approximation errors are not available. In this paper, we will deeply study the hyperbolic cross approximation in Fourier cosine analyses and give a precise asymptotic formula of approximation error of hyperbolic cross truncation. Although our research is in the setting of stochastic processes, our results are also new for deterministic functions.

In order to obtain asymptotic formulas of approximation errors, we first decompose stochastic process *ξ* on [0,1]^2^ into a sum of three terms *ξ* = *P*_*ξ*_ + *ξ*_2_ + *ξ*_3_, where *P*_*ξ*_ is a stochastic polynomial determined by partial derivatives of *ξ* at vertexes of [0,1]^2^, *ξ*_2_ is determined by partial derivative values of *ξ* on the boundary of [0,1]^2^ and is a sum of four univariate stochastic processes with simple polynomial factors, and *ξ*_3_ is a bivariate stochastic process whose partial derivatives vanish on the boundary of [0,1]^2^.

Secondly, based on the above decomposition, we show that Fourier cosine coefficients of a bivariate stochastic process *ξ* on [0,1]^2^ can be approximated asymptotically by a combination of partial derivatives of *ξ* at vertexes of [0,1]^2^ and univariate cosine coefficients on the boundary of [0,1]^2^.

Thirdly, for hyperbolic cross approximation, we give the following precise result: if *ξ* is a stochastic process on [0,1]^2^ with smoothness index *l* ≥ 2, then its hyperbolic cross truncations *S*_*N*_^(*h*)^(*ξ*) (see (16)) of stochastic Fourier cosine series of *ξ* satisfy the following asymptotic formula:

(2) E[∫[0,1]2(SN(h)(ξ;t)−ξ(t))2dt]=Mlog⁡NN3(1+o(1))(N⟶∞),

where

(3) M=43π8E[(ξ(1,1)(0,0))2+(ξ(1,1)(0,1))2+(ξ(1,1)(1,0))2+(ξ(1,1)(1,1))2],

and *E* is the mathematical expectation.

Finally, based on our decomposition of stochastic processes, we propose Fourier cosine expansions with polynomial factors (see (111)) whose hyperbolic cross truncations are a combination of stochastic algebraic polynomials and stochastic cosine polynomials. When the smoothness index *l* ≥ 3 of the stochastic process, for partial sum approximation, hyperbolic cross approximation, and hyperbolic cross approximation with polynomial factors, the square of their approximation errors are

(4) $$\begin{array}{c} O\left(\frac{1}{N_{c}^{3/2}}\right),\;\;O\left(\frac{\log^{4}N_{c}}{N_{c}^{3}}\right),\;\;o\left(\frac{\log^{6}N_{c}}{N_{c}^{5}}\right), \end{array}$$

respectively, where *N*_*c*_ is the number of Fourier cosine coefficients used in each approximation method. From this, we see that hyperbolic cross approximation with polynomial factors can reconstruct the stochastic process on [0,1]^2^ by using the least Fourier cosine coefficients.

This paper is organized as follows. In Section 2 we recall stochastic calculus and stochastic Fourier cosine series. In Section 3 we give decompositions of stochastic processes. In Section 4 we discuss univariate stochastic Fourier cosine analyses. In Section 5 we give an asymptotic formula of Fourier cosine coefficients for bivariate stochastic processes. In Section 6 we discuss partial sum approximations. In Section 7 we discuss hyperbolic cross approximations. In Section 8, we present the Fourier cosine series with polynomial factors and study its hyperbolic cross approximations.

## 2. Fourier Cosine Series of Stochastic Processes 

We recall some concepts in calculus of stochastic processes and stochastic Fourier cosine series.

### 2.1. Calculus of Stochastic Processes

For a stochastic variable *ξ*, we denote its expectation, second-order moment, and variance by *E*[*ξ*], *E*[*ξ* ^2^], and Var⁡(*ξ*), respectively. If *ξ*(*t*) is a stochastic variable for each *t* ∈ [0,1]^*d*^, then we say *ξ*(*t*) is a stochastic process on [0,1]^*d*^. In this paper, we always assume that a stochastic process *ξ*(*t*) is real-valued and satisfies *E*[*ξ* ^2^(*t*)] < *∞* for each *t*. This ensures that its expectation, variance, and second-order moment always exist. Calculus of stochastic processes is a generalization of classical calculus. Let {*ξ*_*n*_}_1_^*∞*^ be a sequence of stochastic variables and let *ξ* be a stochastic variable. If lim⁡_*n*→*∞*_⁡*E*[|*ξ*_*n*_−*ξ*|^2^] = 0, we say that *ξ* is the limit of {*ξ*_*n*_}_1_^*∞*^. Starting from the concept of the limit, one defines continuity, derivatives, partial derivatives, integrals, and double integrals of stochastic processes [7]. Moreover, Newton-Leibnitz formula in calculus of stochastic processes is as follows. If *ξ* is a differentiable stochastic process on [0,1] and the derivative *ξ*′ is continuous on [0,1], then

(5) $$\begin{array}{c} \overset{1}{\underset{0}{\int }}\xi^{\prime }\left(t\right)\text{d}t=\xi \left(1\right)-\xi \left(0\right). \end{array}$$

Let *ξ*(*t*) be a continuously differentiable stochastic process on [0,1] and let *f*(*t*) be a continuously differentiable deterministic function on [0,1]. Then [7]

(6) (ξ(t)f(t))′=ξ′(t)f(t)+ξ(t)f′(t);∫01ξ(t)f′(t)dt=ξ(1)f(1)−ξ(0)f(0)−∫01ξ′(t)f(t)dt.

Burkardt et al. studied stochastic partial differential equations in [8]. Xiu reviewed the current state-of-the-art of numerical method for stochastic computations in [9].

### 2.2. Fourier Cosine Series

If *ξ*(*t*) is a univariate stochastic process on [0,1] and *E*[∫_0_^1^ *ξ* ^2^(*t*)d*t*] < *∞*, then it can be expanded into the Fourier cosine series

(7) $$\begin{array}{c} \xi \left(t\right)=\overset{\infty }{\underset{n=0}{\sum }}c_{n}\left(\xi \right)\cos\;\pi nt \end{array}$$

in mean square sense; that is,

(8) E[||SN(ξ)−ξ||22] =E[∫01(∑n=0N−1cn(ξ)cos⁡(πnt)−ξ(t))2dt]⟶0as  N⟶∞,

where

(9) c0(ξ)=∫01ξ(t)dt,cn(ξ)=2∫01ξ(t)cos⁡⁡(πnt)dt (n≠0).

The corresponding Parseval identity is

(10) $$\begin{array}{c} E\left[\overset{1}{\underset{0}{\int }}\xi^{2}\left(t\right)\text{d}t\right]=E\left[c_{0}^{2}\left(\xi \right)\right]+\frac{1}{2}\overset{\infty }{\underset{n=1}{\sum }}E\left[c_{n}^{2}\left(\xi \right)\right]. \end{array}$$

If *ξ*(*t*_1_, *t*_2_) is a bivariate stochastic process on [0,1]^2^ and *E*[∫_[0,1]^2^_ *ξ* ^2^(*t*_1_, *t*_2_)d*t*_1_d*t*_2_] < *∞*, then it can be expanded into the Fourier cosine series

(11) $$\begin{array}{c} \xi \left(t_{1},t_{2}\right)=\overset{\infty }{\underset{n_{1},n_{2}=0}{\sum }}c_{n_{1},n_{2}}\left(\xi \right)\cos\left(\pi n_{1}t_{1}\right)\cos\left(\pi n_{2}t_{2}\right) \end{array}$$

in mean square sense, where Fourier sine coefficients

(12) cn1,n2(ξ) =λn1,n2∫[0,1]2ξ(t1,t2)cos⁡(πn1t1)cos⁡(πn2t2)dt1dt2

are stochastic variables and

(13) $$\begin{array}{c} \lambda_{n_{1},n_{2}}=\{\begin{array}{cc} 1, & n_{1}=n_{2}=0,\\ 2, & n_{1}=0,\;\;n_{2}\neq 0\;\;\text{or}\;\;n_{2}=0,\;n_{1}\neq 0,\\ 4, & n_{1}\neq 0,\;\;n_{2}\neq 0. \end{array} \end{array}$$

The corresponding Parseval identity holds:

(14) $$\begin{array}{c} E\left[\overset{}{\underset{{\left[0,1\right]}^{2}}{\int }}\xi^{2}\left(t_{1},t_{2}\right)\text{d}t_{1}\text{d}t_{2}\right]=\overset{\infty }{\underset{n_{1},n_{2}=0}{\sum }}\frac{E\left[c_{n_{1},n_{2}}^{2}\left(\xi \right)\right]}{\lambda_{n_{1},n_{2}}}. \end{array}$$

### 2.3. Partial Sums and Hyperbolic Cross Truncations

Let *ξ* be a stochastic process on [0,1]^2^. Partial sums of its Fourier cosine series are

(15) SN(r)(ξ;t1,t2)=∑n1,n2=0N−1cn1,n2(ξ)cos⁡(πn1t1)cos⁡(πn2t2)(N∈Z+).

The number of Fourier cosine coefficients in the partial sum *S*_*N*_^(*r*)^(*ξ*) is *N* ^2^.

Hyperbolic cross truncations of its Fourier cosine series are

(16) SN(h)(ξ;t1,t2) =∑n1=0N−1cn1,0(ξ)cos⁡(πn1t2)  +∑n2=1N−1  ∑n1=0[(N−1)/n2]−1cn1,n2(ξ) cos⁡(πn1t1)cos⁡(πn2t2),

where [·] means the integral part. The number of Fourier cosine coefficients in the hyperbolic cross truncation *S*_*N*_^(*h*)^(*ξ*) is of order *N*log⁡*N*. The hyperbolic cross approximations have been widely used in multivariate function approximation [1, 3–5].

### 2.4. Some Notations

For convenience, we denote vertexes of the unit square [0,1]^2^ by {0,1}^2^, the boundary of [0,1]^2^ by ∂([0,1]^2^).

For a bivariate stochastic process *ξ*(*t*_1_, *t*_2_), denote its mixed derivative ∂^*l*_1_+*l*_2_^ *ξ*/∂*t*_1_^*l*_1_^∂*t*_2_^*l*_2_^ by *ξ* ^(*l*_1_,*l*_2_)^. The notation *C*_*s*_([0,1]^2^) represents the set of continuous stochastic processes on [0,1]^2^. If *ξ* ^(*l*,*l*)^ ∈ *C*_*s*_([0,1]^2^), then we say *ξ* has the smoothness index *l* on [0,1]^2^.

Let {*a*_*n*_} and {*b*_*n*_} be two sequences. If *K*_1_ | *a*_*n*_ | ≤|*b*_*n*_ | ≤*K*_2_ | *a*_*n*_| for any *n*, then we say *a*_*n*_ ~ *b*_*n*_; if |*a*_*n*_ | ≤*K*_3_ | *b*_*n*_| for any *n*, then we say *a*_*n*_ = *O*(*b*_*n*_); here *K*_*i*_ are constants independent of *n*. If *a*_*n*_ → 0, *b*_*n*_ → 0, and *a*_*n*_/*b*_*n*_ → 0 as *n* → *∞*, then we say *a*_*n*_ = *o*(*b*_*n*_). If *a*_*n*_1_,*n*_2__ → 0 and *b*_*n*_1_,*n*_2__ → 0, and *a*_*n*_1_,*n*_2__/*b*_*n*_1_,*n*_2__ → 0 as $||n||=\sqrt{n_{1}^{2}+n_{2}^{2}}\to \infty$, then we say *a*_*n*_1_,*n*_2__ = *o*(*b*_*n*_1_,*n*_2__).

## 3. Decomposition of Stochastic Processes 

In order to study stochastic Fourier cosine series, we give a decomposition of stochastic processes. Although this decomposition is given in the setting of stochastic processes, it is also new for deterministic functions.

Let *ξ* be a bivariate stochastic process on [0,1]^2^ and *ξ* ^(*l*,*l*)^ ∈ *C*_*s*_([0,1]^2^) for some *l* ≥ 1. First, based on a fundamental polynomial *p*(*t*) = *t* ^2^/2 − 1/6, we construct a stochastic polynomial *P*_*ξ*_ as follows:

(17) Pξ(t1,t2)=ξ(1,1)(0,0)p(1−t1)p(1−t2) −ξ(1,1)(0,1)p(1−t1)p(t2) −ξ(1,1)(1,0)p(t1)p(1−t2) +ξ(1,1)(1,1)p(t1)p(t2).

This stochastic polynomial is determined by partial derivatives of *ξ* at vertexes {0,1}^2^.

Denote

(18) $$\begin{array}{c} \xi_{1}\left(t_{1},t_{2}\right)=\xi \left(t_{1},t_{2}\right)-P_{\xi }\left(t_{1},t_{2}\right). \end{array}$$

The following is clear.

**Proposition 1 .**

Let *ξ* be a bivariate stochastic process on [0,1]^2^ with smoothness index *l* ≥ 1 and *ξ*_1_ be stated in (18). Then

(19) $$\begin{array}{c} \xi_{1}^{\left(1,1\right)}\left(0,0\right)=\xi_{1}^{\left(1,1\right)}\left(0,1\right)=\xi_{1}^{\left(1,1\right)}\left(1,0\right)=\xi_{1}^{\left(1,1\right)}\left(1,1\right)=0. \end{array}$$

Now we define

(20) ξ2(t1,t2)=−ξ1(1,0)(0,t2)p(1−t1) +ξ1(1,0)(1,t2)p(t1)−ξ1(0,1)(t1,0)p(1−t2) +ξ1(0,1)(t1,1)p(t2),

(21) $$\begin{array}{c} \xi_{3}\left(t_{1},t_{2}\right)=\xi_{1}\left(t_{1},t_{2}\right)-\xi_{2}\left(t_{1},t_{2}\right), \end{array}$$

and we derive the following.

**Proposition 2 .**

Let *ξ* be a bivariate stochastic process on [0,1]^2^ with smoothness index *l* ≥ 1 and *ξ*_3_ be stated in (21). Then

(22) $$\begin{array}{c} \xi_{3}^{\left(1,1\right)}\left(t_{1},t_{2}\right)=0,\;\left(t_{1},t_{2}\right)\in \partial \left({\left[0,1\right]}^{2}\right). \end{array}$$

**Proof**

Consider the bottom side 0 ≤ *t*_1_ ≤ 1, *t*_2_ = 0 of the square [0,1]^2^. Using Proposition 1, it follows from (20) that *ξ*_2_^(1,1)^(*t*_1_, 0) = *ξ*_1_^(1,1)^(*t*_1_, 0)*p*′(1) = *ξ*_1_^(1,1)^(*t*_1_, 0)  (0 ≤ *t*_1_ ≤ 1). Therefore, by *ξ*_3_ = *ξ*_1_ − *ξ*_2_, we get *ξ*_3_^(1,1)^(*t*_1_, *t*_2_) = 0  (0 ≤ *t*_1_ ≤ 1, *t*_2_ = 0). Similarly, *ξ*_3_^(1,1)^ vanishes on other sides of the square [0,1]^2^.

From (18) and (21), we get a decomposition of stochastic processes on [0,1]^2^ as follows.

Let *ξ* be a stochastic process on [0,1]^2^ and *ξ* ^(*l*,*l*)^ ∈ *C*_*s*_([0,1]^2^) for some *l* ≥ 1. Then the decomposition

(23) $$\begin{array}{c} \xi =P_{\xi }+\xi_{2}+\xi_{3} \end{array}$$

holds, where *P*_*ξ*_, *ξ*_2_, and *ξ*_3_ are stated in (17), (20), and (21), respectively.

## 4. Univariate Stochastic Fourier Cosine Series 

Suppose that *ξ* is a stochastic process on [0,1] and the derivative *ξ* ^(*l*)^ ∈ *C*_*s*_([0,1]) for some *l* ≥ 2. Its Fourier cosine coefficients are as follows:

(24) $$\begin{array}{c} c_{n}\left(\xi \right)=2\overset{1}{\underset{0}{\int }}\xi \left(t\right)\cos\left(\pi nt\right)\text{d}t=g_{n}+r_{n}, \end{array}$$

where

(25) gn=−2(nπ)2(ξ′(0)−(−1)nξ′(1)),rn=−2(nπ)2∫01ξ′′(t)cos⁡(πnt)dt.

Since the expectation and the integral can be exchanged and *E*[*ξ*′′(*t*)] ∈ *C*([0,1]), using the Riemann-Lebesgue lemma [10–12], we get

(26) $$\begin{array}{c} E\left[r_{n}\right]=-\frac{2}{{\left(n\pi \right)}^{2}}\overset{1}{\underset{0}{\int }}E\left[\xi^{\prime \prime }\left(t\right)\right]\cos\left(\pi nt\right)\text{d}t=o\left(\frac{1}{n^{2}}\right). \end{array}$$

From (25),

(27) $$\begin{array}{c} r_{n}^{2}=\frac{4}{{\left(n\pi \right)}^{4}}\overset{}{\underset{{\left[0,1\right]}^{2}}{\int }}\xi^{\prime \prime }\left(t\right)\xi^{\prime \prime }\left(s\right)\cos\left(\pi nt\right)\cos\left(\pi ns\right)\text{d}t\;\text{d}s. \end{array}$$

Again, by the Riemann-Lebesgue lemma, we get

(28) E[rn2] =4(nπ)4∫[0,1]2E[ξ′′(t)ξ′′(s)]cos⁡(πnt)cos⁡(πns)dt ds =o(1n4).

Since Var⁡(*r*_*n*_) ≤ *E*[*r*_*n*_^2^], we have Var⁡(*r*_*n*_) = *o*(1/*n* ^4^). By (24) and (28), we get

(29) E[cn2(ξ)]=E[gn2+2gnrn+rn2]=E[gn2]+2E[gnrn]+o(1n4).

By the Schwarz inequality,

(30) $$\begin{array}{c} \left|E\left[g_{n}r_{n}\right]\right|\leq {\left(E[g_{n}^{2}]\right)}^{1/2}{\left(E[r_{n}^{2}]\right)}^{1/2}=o\left(\frac{1}{n^{4}}\right). \end{array}$$

Therefore, we have

(31) E[c2n2(ξ)]+E[c2n+12(ξ)] =12(nπ)4E[(ξ′(0))2+(ξ′(1))2]+o(1n4).

By the Parseval identity, the partial sums of its Fourier cosine series *S*_*N*_(*ξ*; *t*) = ∑_*k*=0_^*N*−1^ *c*_*k*_(*ξ*)cos⁡(*πnt*) satisfy

(32) E[||SN(ξ)−ξ||22]=E[∫01(SN(ξ;t)−ξ(t))2dt]=12∑n=N∞E[cn2(ξ)]=23π4N3E[(ξ′(0))2+(ξ′(1))2] +o(1N3).

From this, we deduce that *E*[||*S*_*N*_(*ξ*)−*ξ*||_2_^2^] = *o*(1/*N* ^3^) if and only if *ξ*′(0) = *ξ*′(1) = 0.

## 5. Asymptotic Representations of Bivariate Fourier Cosine Coefficients 

Suppose that *ξ* is a bivariate stochastic process on [0,1]^2^ and *ξ* ^(*l*,*l*)^ ∈ *C*_*s*_([0,1]^2^) for some *l* ≥ 2. We expand *ξ* into the Fourier cosine series

(33) $$\begin{array}{c} \xi \left(t_{1},t_{2}\right)=\overset{\infty }{\underset{n_{1},n_{2}=0}{\sum }}c_{n_{1},n_{2}}\left(\xi \right)\cos\left(\pi n_{1}t_{1}\right)\cos\left(\pi n_{2}t_{2}\right) \end{array}$$

and Fourier coefficients

(34) cn1,n2(ξ) =λn1,n2∫[0,1]2ξ(t1,t2)cos⁡(πn1t1)cos⁡(πn2t2)dt1 dt2,

where *λ*_*n*_1_,*n*_2__ is stated in (13).

Based on the decomposition (23) of bivariate stochastic processes, we have

(35) $$\begin{array}{c} c_{n_{1},n_{2}}\left(\xi \right)=c_{n_{1},n_{2}}\left(P_{\xi }\right)+c_{n_{1},n_{2}}\left(\xi_{2}\right)+c_{n_{1},n_{2}}\left(\xi_{3}\right). \end{array}$$

In order to obtain the asymptotic representation of Fourier cosine coefficients *c*_*n*_1_,*n*_2__(*ξ*), we will precisely compute the first two terms and estimate the expectation and variance of the last term on the right-hand side of (35) as follows.

(i) For the first term *c*_*n*_1_,*n*_2__(*P*_*ξ*_), by the representation (17) of stochastic polynomial *P*_*ξ*_, we get that, for *n*_1_ ≠ 0 and *n*_2_ ≠ 0,

(36) $$\begin{array}{c} c_{n_{1},n_{2}}\left(P_{\xi }\right)=\frac{4}{\pi^{4}n_{1}^{2}n_{2}^{2}}\eta_{n_{1},n_{2}}, \end{array}$$

where

(37) ηn1,n2=ξ(1,1)(0,0)−(−1)n2ξ(1,1)(0,1) −(−1)n1ξ(1,1)(1,0)+(−1)n1+n2ξ(1,1)(1,1)

is an algebraic sum of values of *ξ* ^(1,1)^ at {0,1}^2^ and signs for addition and subtraction are determined by odevity of *n*_1_ and *n*_2_. Since ∫_0_^1^ *p*(*t*)d*t* = 0, we get *c*_0,0_(*P*_*ξ*_) = *c*_*n*_1_,0_(*P*_*ξ*_) = *c*_0,*n*_2__(*P*_*ξ*_) = 0.

(ii) For the second term *c*_*n*_1_,*n*_2__(*ξ*_2_), by (20), we know that *ξ*_2_ is the sum of products of separated variables.

Denote

(38) φ1=ξ1(1,0)(0,·),  φ2=ξ1(1,0)(1,·),φ3=ξ1(1,0)(·,0),  φ4=ξ1(1,0)(·,1).

The Fourier cosine coefficients of *ξ*_2_ are equal to

(39) cn1,n2(ξ2)=−cn2(φ1)cn1(p(1−·))+cn2(φ2)cn1(p) −cn1(φ3)cn2(p(1−·))+cn1(φ4)cn2(p),

where each *c*_*n*_(*φ*_*i*_) is the Fourier cosine coefficient of the univariate stochastic process *φ*_*i*_ and each *c*_*n*_(*p*) and each *c*_*n*_(*p*(1 − ·)) are both Fourier cosine coefficients of univariate deterministic functions *p* and *p*(1 − ·). A direct computation shows that, for *n*_1_ ≠ 0 and *n*_2_ ≠ 0,

(40) cn1,n2(ξ2)=2π2n12(−cn2(φ1)+(−1)n1cn2(φ2)) +2π2n22(−cn1(φ3)+(−1)n2cn1(φ4)).

(iii) For the last term *c*_*n*_1_,*n*_2__(*ξ*_3_), using the integration by parts, we deduce that

(41) cn1,n2(ξ3)=1π2n1n2 ×∫01(∫01ξ3(1,1)(t1,t2)sin⁡(πn1t1)dt1) ×sin(πn2t2)dt2.

By Proposition 2, *ξ*_3_^(1,1)^(0, *t*_2_) = *ξ*_3_^(1,1)^(1, *t*_2_) = 0  (0 ≤ *t*_2_ ≤ 1), the interior integral is equal to

(42) $$\begin{array}{c} \frac{1}{\pi n_{1}}\overset{1}{\underset{0}{\int }}\xi_{3}^{\left(2,1\right)}\left(t_{1},t_{2}\right)\cos\left(\pi n_{1}t_{1}\right)\text{d}t_{1}. \end{array}$$

So

(43) cn1,n2(ξ3)=1π3n12n2 ×∫01(∫01ξ3(2,1)(t1,t2)sin(πn2t2)dt2)×cos⁡(πn1t1)dt1.

By *ξ*_3_^(1,1)^(*t*_1_, 0) = 0  (0 ≤ *t*_1_ ≤ 1), we have *ξ*_3_^(2,1)^(*t*_1_, 1) = 0  (0 ≤ *t*_1_ ≤ 1). This implies that

(44) cn1,n2(ξ3)=4(n1π)2(n2π)2 ×∫[0,1]2ξ3(2,2)(t1,t2)cos⁡⁡(πn1t1)×cos⁡⁡(πn2t2)dt1 dt2,

and so

(45) cn1,n22(ξ3)=16(n1π)4(n2π)4 ×∫[0,1]4ξ3(2,2)(t1,t2)ξ3(2,2)(s1,s2)×cos⁡(πn1t1)cos⁡(πn2t2)cos⁡(πn1s1)×cos⁡(πn2s2)dt1 dt2 ds1 ds2.

Taking expectations on these two equations, we get

(46) E[cn1,n2(ξ3)]=4(n1π)2(n2π)2 ×∫[0,1]2E[ξ3(2,2)(t1,t2)]cos⁡(πn1t1)×cos⁡(πn2t2)dt1dt2,E[cn1,n22(ξ3)]=16(n1π)4(n2π)4 ×∫[0,1]4E[ξ3(2,2)(t1,t2)ξ3(2,2)(s1,s2)]×cos⁡(πn1t1)cos⁡(πn2t2)cos⁡(πn1s1)×cos⁡(πn2s2)dt1 dt2 ds1 ds2.

It follows from *ξ*_3_^(2,2)^ ∈ *C*_*s*_([0,1]^2^) that *E*[*ξ*_3_^(2,2)^] is a continuous function on [0,1]^2^ and *E*[*ξ*_3_^(2,2)^(*t*_1_, *t*_2_)*ξ*_3_^(2,2)^(*s*_1_, *s*_2_)] is a continuous on [0,1]^4^. By the Riemann-Lebesgue lemma, we have

(47) $$\begin{array}{c} E\left[c_{n_{1},n_{2}}\left(\xi_{3}\right)\right]=o\left(\frac{1}{n_{1}^{2}n_{2}^{2}}\right),\;\;E\left[c_{n_{1},n_{2}}^{2}\left(\xi_{3}\right)\right]=o\left(\frac{1}{n_{1}^{4}n_{2}^{4}}\right),\\ \mathrm{Var}\left(c_{n_{1},n_{2}}\left(\xi \right)\right)=o\left(\frac{1}{n_{1}^{4}n_{2}^{4}}\right) \end{array}$$

since Var⁡(*c*_*n*_1_,*n*_2__(*ξ*_3_)) ≤ *E*[*c*_*n*_1_,*n*_2__^2^(*ξ*_3_)].

From (i), (ii), and (iii), we get

(48) cn1,n2(ξ)=4π4n12n22ηn1,n2 +2π2n12(−cn2(φ1)+(−1)n1cn2(φ2)) +2π2n22(−cn1(φ3)+(−1)n2cn1(φ4)) +cn1,n2(ξ3),

and the error *c*_*n*_1_,*n*_2__(*ξ*) satisfies (47), where *η*_*n*_1_,*n*_2__ is stated in (37) and each *φ*_*i*_ is stated in (38).

Now we further estimate these four univariate Fourier cosine coefficients *c*_*n*_(*φ*_*i*_).

**Lemma 3 .**

Let each *φ*_*i*_ be stated as in (38). Then Fourier cosine coefficients *c*_*n*_(*φ*_*i*_) satisfy

(49) E[cn(φi)]=o(1n2),  E[cn2(φi)]=o(1n4),(i=1,2,3,4).

**Proof**

By similarity, we only prove the case *i* = 1, since *φ*_1_ = *ξ*_1_^(1,0)^(0, ·). By Proposition 1 and *ξ*_1_ = *ξ* − *P*_*ξ*_, we have *φ*_1_′(0) = *φ*_1_′(1) = 0. From this, we deduce that Fourier cosine coefficients satisfy

(50) $$\begin{array}{c} E\left[c_{n}\left(\varphi_{1}\right)\right]=o\left(\frac{1}{n^{2}}\right),\;\;E\left[c_{n}^{2}\left(\varphi_{1}\right)\right]=o\left(\frac{1}{n^{4}}\right). \end{array}$$

We will deduce these asymptotic representations of Fourier cosine coefficients.

**Theorem 4 .**

Let *ξ* be a stochastic process on [0,1]^2^ and *ξ* ^(*l*,*l*)^ ∈ *C*_*s*_([0,1]^2^) for some *l* ≥ 2, and let *η*_*n*_1_,*n*_2__ and *φ*_*i*_ be stated in (37) and (38), respectively. Then

(i) * for n*_1_ → *∞*, *c*_*n*_1_,*n*_2__(*ξ*) = *b*_*n*_1_,*n*_2__ + *τ*_*n*_1_,*n*_2__,* where*
  (51) bn1,n2=4π4n12n22ηn1,n2 +2π2n12(−cn2(φ1)+(−1)n1cn2(φ2)),
 and *τ*_*n*_1_,*n*_2__ satisfies
  (52) $$\begin{array}{c} E\left[\tau_{n_{1},n_{2}}\right]=o\left(\frac{1}{n_{1}^{2}}\right)\frac{1}{n_{2}^{2}},\;\;E\left[\tau_{n_{1},n_{2}}^{2}\right]=o\left(\frac{1}{n_{1}^{4}}\right)\frac{1}{n_{2}^{4}},\\ \mathrm{Var}\left(\tau_{n_{1},n_{2}}\right)=o\left(\frac{1}{n_{1}^{2}}\right)\frac{1}{n_{2}^{2}}; \end{array}$$
(ii) * for n*_2_ → *∞*, $c_{n_{1},n_{2}}(\xi )={\tilde{b}}_{n_{1},n_{2}}+{\tilde{\tau }}_{n_{1},n_{2}}$, where
  (53) b~n1,n2=4π4n12n22ηn1,n2 +2π2n22(−cn1(φ3)+(−1)n2cn1(φ4)),
 and ${\tilde{\tau }}_{n_{1},n_{2}}$ satisfies
  (54) $$\begin{array}{c} E\left[{\tilde{\tau }}_{n_{1},n_{2}}\right]=o\left(\frac{1}{n_{2}^{2}}\right)\frac{1}{n_{1}^{2}},\;\;E\left[{\tilde{\tau }}_{n_{1},n_{2}}^{2}\right]=o\left(\frac{1}{n_{2}^{4}}\right)\frac{1}{n_{1}^{4}},\\ \mathrm{Var}\left({\tilde{\tau }}_{n_{1},n_{2}}\right)=o\left(\frac{1}{n_{2}^{2}}\right)\frac{1}{n_{1}^{2}}; \end{array}$$
(iii) * for n*_1_ → *∞* and *n*_2_ → *∞*, *c*_*n*_1_,*n*_2__(*ξ*) = (4/*π* ^2^ *n*_1_^2^ *n*_2_^2^)*η*_*n*_1_,*n*_2__ + *τ*_*n*_1_,*n*_2__* and *τ*_*n*_1_,*n*_2__* satisfies
  (55) $$\begin{array}{c} E\left[\tau_{n_{1},n_{2}}^{*}\right]=o\left(\frac{1}{n_{1}^{2}n_{2}^{2}}\right),\;\;E\left[\tau_{n_{1},n_{2}}^{*2}\right]=o\left(\frac{1}{n_{1}^{4}n_{2}^{4}}\right),\\ \mathrm{Var}\left(\tau_{n_{1},n_{2}}^{*}\right)=o\left(\frac{1}{n_{1}^{4}n_{2}^{4}}\right). \end{array}$$

**Proof**

When *n*_1_ → *∞*, denote

(56) $$\begin{array}{c} \tau_{n_{1},n_{2}}=\frac{2}{{\left(n\pi \right)}^{2}}\left(-c_{n_{1}}\left(\varphi_{3}\right)+{\left(-1\right)}^{n_{2}}c_{n_{1}}\left(\varphi_{4}\right)\right)+c_{n_{1},n_{2}}\left(\xi_{3}\right). \end{array}$$

By (48), *c*_*n*_1_,*n*_2__(*ξ*) = *b*_*n*_1_,*n*_2__ + *τ*_*n*_1_,*n*_2__. Using Lemma 3 and (47), we deduce that *E*[*τ*_*n*_1_,*n*_2__] = *o*(1/*n*_1_^2^)(1/*n*_2_^2^) and

(57) E[τn1,n22]≤K(1n22E[cn12(φ3)+cn12(φ4)]+E[cn1,n22(ξ3)])=o(1n14)1n24.

So we get (i). Similarly, we can get (ii).

From this, we can give asymptotic representations of expectation, second-order moment, and variance of Fourier cosine coefficients.

**Corollary 5 .**

Under conditions of Theorem 4, we have

(i) * for n*_1_ → *∞* and *n*_2_ ≠ 0,
  (58) E[cn1,n2(ξ)]=E[bn1,n2]+o(1n12)1n22,E[cn1,n22(ξ)]=E[bn1,n22]+o(1n14)1n24,Var⁡(cn1,n2(ξ))=Var⁡(bn1,n2)+o(1n14)1n24,
 where *b*_*n*_1_,*n*_2__ is stated in (51) and “*o*” is uniform for *n*_2_;
(ii) * for n*_2_ → *∞* and *n*_1_ ≠ 0,
  (59) E[cn1,n2(ξ)]=E[b~n1,n2]+o(1n22)1n12,E[cn1,n22(ξ)]=E[b~n1,n22]+o(1n24)1n14,Var⁡(cn1,n2(ξ))=Var⁡(b~n1,n2)+o(1n24)1n14,
 where ${\tilde{b}}_{n_{1},n_{2}}$ is stated in (53) and “*o*” is uniform for *n*_1_.

**Proof**

By similarity, we only prove (58).

From Theorem 4 (i), we deduce that, for *n*_1_ → *∞*,   *n*_2_ ≠ 0,

(60) E[cn1,n22(ξ)]=E[bn1,n22]+2E[bn1,n2τn1,n2]+o(1n14)1n24,|E[bn1,n2λn1,n2]|≤E[bn1,n22]·E[τn1,n22]=o(1n14)1n24.

So we get (58).

From Corollary 5 (iii), we know that

(61) $$\begin{array}{c} E\left[c_{n_{1},n_{2}}\left(\xi \right)\right]\sim \frac{1}{n_{1}^{2}n_{2}^{2}},\;\;E\left[c_{n_{1},n_{2}}^{2}\left(\xi \right)\right]\sim \frac{1}{n_{1}^{4}n_{2}^{4}}. \end{array}$$

These results cannot be improved as smoothness index *l* increases.

## 6. Approximation of Partial Sums 

Let *ξ* be a stochastic process on [0,1]^2^ and *ξ* ^(*l*,*l*)^ ∈ *C*_*s*_([0,1]^2^) for some *l* ≥ 2. We expand *ξ* into the Fourier cosine series:

(62) $$\begin{array}{c} \xi \left(t_{1},t_{2}\right)=\overset{\infty }{\underset{n_{1},n_{2}=0}{\sum }}c_{n_{1},n_{2}}\left(\xi \right)\cos\left(\pi n_{1}t_{1}\right)\cos\left(\pi n_{2}t_{2}\right). \end{array}$$

We give further the asymptotic representation of approximation error of partial sums. The partial sums *S*_*N*_^(*r*)^(*ξ*) of Fourier cosine series (62) are defined as

(63) $$\begin{array}{c} S_{N}^{\left(r\right)}\left(\xi ;t_{1},t_{2}\right)=\overset{N-1}{\underset{n_{1},n_{2}=0}{\sum }}c_{n_{1},n_{2}}\left(\xi \right)\cos\left(\pi n_{1}t_{1}\right)\cos\left(\pi n_{2}t_{2}\right). \end{array}$$

Using the Parseval identity, we get

(64) E[||SN(r)(ξ)−ξ||22]=(∑n1,n2=0∞−∑n1,n2=0N−1)E[cn1,n22(ξ)]λn1,n2=14(∑n1,n2=1∞−∑n1,n2=1N−1)E[cn1,n22(ξ)] +12∑n1=N∞E[cn1,02(ξ)] +12∑n2=N∞E[c0,n22(ξ)]=:  PN+QN+RN.

(i) We compute *P*_*N*_. The first term *P*_*N*_ on the right-hand side of the formula (64) can be decomposed into three sums
  (65) $$\begin{array}{c} P_{N}=\frac{1}{4}\left(P_{N}^{1}+P_{N}^{2}+P_{N}^{3}\right), \end{array}$$
 where
  (66) PN1=∑n2=1  N−1∑n1=N∞E[cn1,n22(ξ)],PN2=∑n1=1  N−1∑n2=N∞E[cn1,n22(ξ)],PN3=∑n1,n2=N∞E[cn1,n22(ξ)].

First, we consider the interior sum of *P*_*N*_^1^ with *N* = 2*k*,

(67) ∑n1=2k∞E[cn1,n22(ξ)]=∑n1=k∞E[c2n1,n22(ξ)] +∑n1=k∞E[c2n1+1,n22(ξ)]=:Xk,n2+Yk,n2.

By Corollary 5 (i), we deduce that

(68) Xk,n2=∑n1=k∞(12π2n12)2E[(bn2(1))2]+o(1k3)1n24,Yk,n2=∑n1=k∞(12π2n12)2E[(bn2(2))2]+o(1k3)1n24,

where “*o*” is uniform for *n*_2_ and

(69) bn2(1)=2π2n22(ξ(1,1)(0,0)−ξ(1,1)(1,0)−(−1)n2(ξ(1,1)(0,1)−ξ(1,1)(1,1))) −cn2(φ1)+cn2(φ2),bn2(2)=2π2n22(ξ(1,1)(0,0)+ξ(1,1)(1,0)−(−1)n2(ξ(1,1)(0,1)+ξ(1,1)(1,1))) −cn2(φ1)−cn2(φ2).

Again, by

(70) $$\begin{array}{c} \overset{\infty }{\underset{n_{1}=k}{\sum }}\frac{1}{{\left(2\pi^{2}n_{1}^{2}\right)}^{2}}=\frac{1}{12\pi^{4}k^{3}}+O\left(\frac{1}{k^{4}}\right) \end{array}$$

and (67), we obtain that

(71) ∑n1=2k∞E[cn1,n22(ξ)]=112π4k3E[(bn2(1))2+(bn2(2))2] +o(1k3)1n24,

where “*o*” is uniform for *n*_2_. By the convergence of the series ∑_*n*=1_^*∞*^(1/*n* ^4^) and (65), we have

(72) $$\begin{array}{c} P_{2k}^{1}=\frac{1}{12\pi^{4}k^{3}}\left(\overset{2k-1}{\underset{n_{2}=1}{\sum }}E\left[{\left(b_{n_{2}}^{\left(1\right)}\right)}^{2}+{\left(b_{n_{2}}^{\left(2\right)}\right)}^{2}\right]\right)+o\left(\frac{1}{k^{3}}\right). \end{array}$$

By (69), we deduce that there exists a constant *K*_1_ > 0 such that

(73) |E[(bn2(1))2+(bn2(2))2]| ≤K1E[1n24∑λ∈{0,1}2(ξ(1,1)(λ))2+cn22(φ1)+cn22(φ2)].

Again, by Lemma 3, we have

(74) $$\begin{array}{c} E\left[{\left(b_{n_{2}}^{\left(1\right)}\right)}^{2}+{\left(b_{n_{2}}^{\left(2\right)}\right)}^{2}\right]=O\left(\frac{1}{n_{2}^{4}}\right), \end{array}$$

and so the series ∑_*n*_2_=1_^*∞*^ *E*[(*b*_*n*_2__^(1)^)^2^ + (*b*_*n*_2__^(2)^)^2^] converges, and denote its sum by *A*. So

(75) $$\begin{array}{c} \overset{2k-1}{\underset{n_{2}=1}{\sum }}E\left[{\left(b_{n_{2}}^{\left(1\right)}\right)}^{2}+{\left(b_{n_{2}}^{\left(2\right)}\right)}^{2}\right]=A+o\left(1\right)\;\left(k⟶\infty \right). \end{array}$$

By (72), we get *P*_2*k*_^1^ = (*A*/12*π* ^4^ *k* ^3^) + *o*(1/*k* ^3^). Notice that

(76) $$\begin{array}{c} P_{2k+1}^{1}=P_{2k}^{1}+\overset{\infty }{\underset{n_{1}=2k+1}{\sum }}E\left[c_{n_{1},2k}^{2}\left(\xi \right)\right]-\overset{2k-1}{\underset{n_{2}=1}{\sum }}E\left[c_{2k,n_{2}}^{2}\left(\xi \right)\right] \end{array}$$

and *E*[*c*_*n*_1_,*n*_2__^2^(*ξ*)] = *O*(1/*n*_1_^4^ *n*_2_^4^) (see (61)). We have *P*_2*k*+1_^1^ = *P*_2*k*_^1^ + *o*(1/*k* ^3^). This implies that

(77) $$\begin{array}{c} \;P_{N}^{1}=\frac{2A}{3\pi^{4}N^{3}}+o\left(\frac{1}{N^{3}}\right),\\ \text{where}\;A=\overset{\infty }{\underset{n_{2}=1}{\sum }}E\left[{\left(b_{n_{2}}^{\left(1\right)}\right)}^{2}+{\left(b_{n_{2}}^{\left(2\right)}\right)}^{2}\right]. \end{array}$$

Here *b*_*n*_2__^(1)^ and *b*_*n*_2__^(2)^ are stated in (69).

Similarly,

(78) $$\begin{array}{c} \;P_{N}^{2}=\frac{2B}{3\pi^{4}N^{3}}+o\left(\frac{1}{N^{3}}\right),\\ \text{where}\;B=\overset{\infty }{\underset{n_{1}=1}{\sum }}E\left[{\left({\tilde{b}}_{n_{1}}^{\left(1\right)}\right)}^{2}+{\left({\tilde{b}}_{n_{1}}^{\left(2\right)}\right)}^{2}\right]. \end{array}$$

Here “*o*” is uniform for *n*_1_ and

(79) b~n1(1)=2π2n12(ξ(1,1)(0,0)−ξ(1,1)(0,1)−(−1)n1(ξ(1,1)(1,0)−ξ(1,1)(1,1))) −cn1(φ3)+cn1(φ4),b~n1(2)=2π2n12(ξ(1,1)(0,0)+ξ(1,1)(0,1)−(−1)n1(ξ(1,1)(1,0)+ξ(1,1)(1,1))) −cn1(φ3)−cn1(φ4).

From *E*[*c*_*n*_1_,*n*_2__^2^(*ξ*)] = *O*(1/*n*_1_^4^ *n*_2_^4^), we have *P*_*N*_^3^ = *o*(1/*N* ^3^). Finally, by (65), we get

(80) $$\begin{array}{c} P_{N}=\frac{A+B}{6\pi^{4}N^{3}}+o\left(\frac{1}{N^{3}}\right), \end{array}$$

where *A*, *B* are stated in (77) and (78).

(ii) We compute *Q*_*N*_ and *R*_*N*_ in (64). By the decomposition formula (23),
  (81) $$\begin{array}{c} c_{n_{1},0}\left(\xi \right)=c_{n_{1},0}\left(P_{\xi }\right)+c_{n_{1},0}\left(\xi_{2}\right)+c_{n_{1},0}\left(\xi_{3}\right). \end{array}$$

By (17) and ∫_0_^1^ *p*(*t*)d*t* = 0, we have *c*_*n*_1_,0_(*P*_*ξ*_) = 0. By (39), we have

(82) $$\begin{array}{c} c_{n_{1},0}\left(\xi_{2}\right)=\frac{2}{{\left(\pi n_{1}\right)}^{2}}\left(-c_{0}\left(\varphi_{1}\right)+{\left(-1\right)}^{n_{1}}c_{0}\left(\varphi_{2}\right)\right). \end{array}$$

From this, we get

(83) E[c2n1,02(ξ2)]+E[c2n1+1,02(ξ2)] =12(πn1)4(E[c02(φ1)+c02(φ2)])+o(1n14).

Similar to (47), we have

(84) E[cn1,02(ξ3)]=4(πn1)4 ×∬01E[ξ3(t1,t2)ξ3(s1,t2)] ×cos⁡⁡(πn1t1)cos⁡⁡(πn1s1)dt1 ds1=o(1n14).

Again, by (81), we have

(85) ∑n1=2k∞E[cn1,02(ξ)]=∑n1=k∞(E[c2n1,02(ξ)]+E[c2n1+1,02(ξ)])=16π4k3(E[c02(φ1)+c02(φ2)]+o(1)).

This implies by (64) that

(86) $$\begin{array}{c} Q_{N}=\frac{2C}{3\pi^{4}N^{3}}+o\left(\frac{1}{N^{3}}\right),\\ \text{where}\;\;C=E\left[c_{0}^{2}\left(\varphi_{1}\right)+c_{0}^{2}\left(\varphi_{2}\right)\right]. \end{array}$$

Similarly, we get

(87) $$\begin{array}{c} R_{N}=\frac{2D}{3\pi^{4}N^{3}}+o\left(\frac{1}{N^{3}}\right),\\ \text{where}\;\;D=E\left[c_{0}^{2}\left(\varphi_{3}\right)+c_{0}^{2}\left(\varphi_{4}\right)\right]. \end{array}$$

From this and (80), we get by (64) the following.

**Theorem 6 .**

Let *ξ* be a stochastic process on [0,1]^2^ and *ξ* ^(*l*,*l*)^ ∈ *C*_*s*_([0,1]^2^) for some *l* ≥ 2. Then the partial sums *S*_*N*_(*ξ*) of its Fourier cosine series satisfy

(88) $$\begin{array}{c} E\left[{||S_{N}^{\left(r\right)}(\xi )-\xi ||}_{2}^{2}\right]=\frac{M}{N^{3}}+o\left(\frac{1}{N^{3}}\right), \end{array}$$

where *M* = (1/6*π* ^4^)(*A* + *B* + 4*C* + 4*D*) and *A*, *B*, *C*, *D* are stated in (77), (78), (86), and (87).

## 7. Approximation of Hyperbolic Cross Truncations 

Suppose that *ξ* is a stochastic process on [0,1]^2^ and *ξ* ^(*l*,*l*)^ ∈ *C*_*s*_([0,1]^2^) for some *l* ≥ 2. We consider hyperbolic cross truncations of its Fourier cosine series:

(89) SN(h)(ξ;t1,t2)=∑n1=0N−1cn1,0(ξ)cos⁡(πn1t2) +∑n2=1N−1  ∑n1=0[(N−1)/n2]−1cn1,n2(ξ)cos⁡⁡(πn1t1)×cos⁡(πn2t2).

By the Parseval identity and (61), (86), and (87), we have

(90) E[||SN(h)(ξ)−ξ||22]=14JN+12∑n1=N∞E[cn1,02(ξ)] +14∑n2=N  ∞∑n1=0∞E[cn1,n22(ξ)]=14JN+O(1N3),

where *J*_*N*_ = ∑_*n*_2_=1_^*N*−1^∑_*n*_1_=[(*N*−1)/*n*_2_]_^*∞*^ *E*[*c*_*n*_1_,*n*_2__^2^(*ξ*)]. We rewrite *J*_*N*_ in the form

(91) JN=∑n2=1[(N−1)/2]∑n1=[(N−1)/4n2]∞(E[c2n1,2n22(ξ)+c2n1−1,2n22(ξ)])  +∑n2=1[(N−1)/2]∑n1=[(N−1)/(4n2−2)]∞ ×(E[c2n1,2n2−12(ξ)]+c2n1−1,2n2−12(ξ))  +O(1N3)=JN1+JN2+O(1N3).

We first consider *J*_*N*_^1^. By Corollary 5 (i), we have

(92) E[c2n1,2n22(ξ)] =14π4n14E[(12π2n22ω1−c2n2(φ1)+c2n2(φ2))2]  +o(1n14)1n24,E[c2n1−1,2n22(ξ)] =14π4n14E[(12π2n22ω2−c2n2(φ1)−c2n2(φ2))2]  +o(1n14)1n24,

where “*o*” is uniform for *n*_2_ and

(93) ω1=ξ(1,1)(0,0)−ξ(1,1)(0,1)  −ξ(1,1)(1,0)+ξ(1,1)(1,1)),ω2=ξ(1,1)(0,0)−ξ(1,1)(0,1)  +ξ(1,1)(1,0)−ξ(1,1)(1,1).

Therefore,

(94) ∑n1=[(N−1)/4n2]∞E[c2n1,2n22(ξ)] =(∑n1=[(N−1)/4n2]∞14π4n14)  ×E[(12π2n22ω1−c2n2(φ1)+c2n2(φ2))2]  +o(1N3)1n2.

Here

(95) ∑n1=[(N−1)/4n2]∞14π4n14=16n233π4N3+O(n24N4),E[(12π2n22ω1−cn2(φ1)+cn2(φ2))2] =14π4n24E[ω12]+μn2,

where *μ*_*n*_2__ = −(1/*π* ^2^ *n*_2_^2^)  *E*[*ω*_1_(*c*_*n*_2__(*φ*_1_) − *c*_*n*_2__(*φ*_2_))] + *E*[(*c*_*n*_2__(*φ*_1_) − *c*_*n*_2__(*φ*_2_))^2^].

By using the Schwarz inequality in Probability theory,

(96) |E[ω1(cn2(φ1)−cn2(φ2))]| ≤(E[ω12])1/2(E[(cn2(φ1)−cn2(φ2))2])1/2.

By Lemma 3, *E*[(*c*_*n*_2__(*φ*_1_) − *c*_*n*_2__(*φ*_2_))^2^] ≤ 2*E*[*c*_*n*_2__^2^(*φ*_1_) + *c*_*n*_2__^2^(*φ*_2_)] = *o*(1/*n*_2_^4^). Therefore, *μ*_*n*_2__ = *o*(1/*n*_2_^4^). From this, we have

(97) E[(12π2n22ω1−cn2(φ1)+cn2(φ2))2] =14π4n24E[ω12]+o(1n24).

Substituting (95) and (97) into (94), we get that, for *n*_2_ ≤ *N*,

(98) ∑n1=[(N−1)/4n2]∞E[c2n1,2n22(ξ)]=4E[ω12]3π8N3n2+o(1N3n2).

Similarly, we have

(99) $$\begin{array}{c} \overset{\infty }{\underset{n_{1}=\left[\left(N-1\right)/4n_{2}\right]}{\sum }}E\left[c_{2n_{1}-1,2n_{2}}^{2}\left(\xi \right)\right]=\frac{4E\left[\omega_{2}^{2}\right]}{3\pi^{8}N^{3}n_{2}}+o\left(\frac{1}{N^{3}n_{2}}\right). \end{array}$$

Denote *ω* = (1/2)(*ω*_1_^2^ + *ω*_2_^2^). We get

(100) $$\begin{array}{c} \omega ={\left(\xi^{\left(1,1\right)}\left(0,0\right)-\xi^{\left(1,1\right)}\left(0,1\right)\right)}^{2}+{\left(\xi^{\left(1,1\right)}\left(1,0\right)-\xi^{\left(1,1\right)}\left(1,1\right)\right)}^{2}. \end{array}$$

This implies that

(101) ∑n1=[(N−1)/4n2]∞E[c2n1,2n22(ξ)+c2n1−1,2n22(ξ)]  =8E[ω]3π8N3n2+o(1N3n2).

So we have

(102) JN1=8E[ω]3π8N3∑n2=1[(N−1)/2]1n2+o(1N3)∑n2=1[(N−1)/2]1n2=8log⁡N3π8N3(E[ω]+o(1)).

Similarly, we have

(103) $$\begin{array}{c} J_{N}^{2}=\frac{8\log N}{3\pi^{8}N^{3}}\left(E\left[\tilde{\omega }\right]+o\left(1\right)\right), \end{array}$$

where $\tilde{\omega }={\left(\xi^{\left(1,1\right)}(0,0)+\xi^{\left(1,1\right)}(0,1)\right)}^{2}+{\left(\xi^{\left(1,1\right)}(1,0)+\xi^{\left(1,1\right)}(1,1)\right)}^{2}$. From this and (91), we deduce by (90) that

(104) JN=∑n2=1[(N−1)/2](8E[ω+ω~]3π8N3n2+o(1N3n2))=16log⁡N3π8N3(E[ω∗]+o(1)),

where ω* = (*ξ* ^(1,1)^(0,0))^2^ + (*ξ* ^(1,1)^(0,1))^2^ + (*ξ* ^(1,1)^(1,0))^2^ + (*ξ* ^(1,1)^(1,1))^2^. Finally, we get by (90) that

(105) $$\begin{array}{c} E\left[{||S_{N}^{\left(h\right)}\left(\xi \right)-\xi ||}_{2}^{2}\right]=\frac{4\log N}{3\pi^{8}N^{3}}\left(E\left[\omega^{*}\right]+o\left(1\right)\right). \end{array}$$

So we get the following.

**Theorem 7 .**

Suppose that *ξ* is a stochastic process on [0,1]^2^ and *ξ* ^(*l*,*l*)^ ∈ *C*_*s*_([0,1]^2^) for some *l* ≥ 2. Let *S*_*N*_^(*h*)^(*ξ*) be the hyperbolic cross truncations of its Fourier cosine series, which is stated in (89). Then the following asymptotic formula holds:

(106) $$\begin{array}{c} E\left[{||S_{N}^{\left(h\right)}\left(\xi \right)-\xi ||}_{2}^{2}\right]=\tilde{M}\frac{\log N}{N^{3}}\left(1+o\left(1\right)\right), \end{array}$$

where $\tilde{M}=\left(4/3\pi^{8}\right)E[{\left(\xi^{\left(1,1\right)}(0,0)\right)}^{2}+{\left(\xi^{\left(1,1\right)}(0,1)\right)}^{2}+{\left(\xi^{\left(1,1\right)}(1,0)\right)}^{2}+{{(\xi }^{\left(1,1\right)}(1,1))}^{2}]$.

From Theorem 7, we know that *E*[||*S*_*N*_^(*h*)^(*ξ*)−*ξ*||_2_^2^] ~ (log⁡*N*/*N* ^3^). This result cannot be improved as the smoothness index *l* increases.

By (89), the number of Fourier cosine coefficients in hyperbolic cross truncation *S*_*N*_^(*h*)^(*ξ*) is

(107) $$\begin{array}{c} N_{c}=N+\overset{N-1}{\underset{n_{1}=1}{\sum }}\left[\frac{N-1}{n_{1}}\right]\sim N\log N \end{array}$$

and log⁡*N*_*c*_ ~ log⁡*N*. From this, we get

(108) $$\begin{array}{c} E\left[{||S_{N}^{\left(h\right)}\left(\xi \right)-\xi ||}_{2}^{2}\right]\sim \frac{\log^{4}N_{c}}{N_{c}^{3}}. \end{array}$$

Since the number of Fourier cosine coefficients in the partial sum *S*_*N*_^(*r*)^(*ξ*) is *N*_*c*_ = *N* ^2^, by Theorem 6, we have

(109) $$\begin{array}{c} E\left[{||S_{N}^{\left(r\right)}\left(\xi \right)-\xi ||}_{2}^{2}\right]\sim \frac{1}{N_{c}^{3/2}}. \end{array}$$

## 8. Approximation of Hyperbolic Cross Truncations with Polynomial Factors

In order to use the least Fourier cosine coefficients to reconstruct stochastic processes, we introduce the Fourier cosine expansion with polynomial factors. Suppose that *ξ* is a stochastic process on [0,1]^2^ and *ξ* ^(*l*,*l*)^ ∈ *C*_*s*_([0,1]^2^) for some *l* ≥ 3. By using notation (38), the decomposition (23) can be rewritten in the form

(110) ξ(t1,t2)=Pξ(t1,t2)+ξ2(t1,t2)+ξ3(t1,t2)=Pξ(t1,t2)−φ1(t2)p(1−t1)+φ2(t2)p(t1) −φ3(t1)p(1−t2)+φ4(t1)p(t2)+ξ3(t1,t2).

In this decomposition, we expand each *φ*_*i*_ into univariate Fourier cosine series and expand *ξ*_3_(*t*_1_, *t*_2_) into bivariate Fourier cosine series. Finally, we obtain the Fourier cosine expansion of *ξ* with polynomial factors:

(111) ξ(t1,t2)=Pξ(t1,t2) +(∑n=0∞cn(φ1)cos⁡(πnt2)) ×(−p(1−t1))+(∑n=0∞cn(φ2)cos⁡⁡(πnt2))p(t1) +(∑n=0∞cn(φ3)cos⁡⁡(πnt1)) ×(−p(1−t2))+(∑n=0∞cn(φ4)cos⁡⁡(πnt1))p(t2) +∑n1,n2=0∞cn1,n2(ξ3)cos⁡(πn1t1)cos⁡(πn2t2),

where *p*(*t*) = (*t* ^2^/2) − (1/6). Notice that *φ*_1_(*t*_2_) = *ξ*_1_^(1,0)^(0, *t*_2_) and *ξ*_1_^(3,3)^ ∈ *C*_*s*_([0,1]^2^), where *ξ*_1_ = *ξ* − *P*_*ξ*_. Using Proposition 1, we have *φ*_1_′′′ ∈ *C*_*s*_([0,1]) and *φ*_1_′(0) = *φ*_1_′(1) = 0, and so

(112) $$\begin{array}{c} c_{n}\left(\varphi_{1}\right)=\frac{2}{{\left(n\pi \right)}^{3}}\overset{1}{\underset{0}{\int }}\varphi_{1}^{\prime \prime \prime }\left(t\right)\sin\left(\pi nt\right)\text{d}t. \end{array}$$

Therefore, we have *E*[*c*_*n*_(*φ*_1_)] = *o*(1/*n* ^3^) and

(113) E[cn2(φ1)]=4(nπ)6 ×∫[0,1]2E[φ1′′′(t)φ1′′′(s)]sin⁡(πnt)×sin⁡(πns)dt ds.

Similarly, for *c*_*n*_(*φ*_2_),*c*_*n*_(*φ*_3_), and *c*_*n*_(*φ*_4_), we get

(114) E[cn(φi)]=o(1n3),  E[cn2(φi)]=o(1n6)(i=1,2,3,4).

From this, we see that these univariate Fourier coefficients decay fast.

Now we show that bivariate Fourier cosine coefficients *c*_*n*_1_,*n*_2__(*h*_3_) decay fast. By *ξ* ^(*l*,*l*)^ ∈ *C*_*s*_([0,1]^3^)  (*l* ≥ 3) and Proposition 2, using the integration by parts, we get

(115) E[cn1,n2(ξ3)]=4(n1π)3(n2π)3 ×∫[0,1]2E[ξ3(3,3)(t1,t2)]sin(πn1t1)×sin(πn2t2)dt1 dt2,E[cn1,n22(ξ3)]=16(n1π)6(n2π)6 ×∫[0,1]4E[ξ3(3,3)(t1,t2)ξ3(3,3)(s1,s2)]×sin(πn1t1)sin⁡(πn2t2)×sin⁡(πn1s1)×sin(πn2s2)dt1 dt2ds1ds2.

So we get

(116) E[cn1,n2(ξ3)]=o(1n13n23),E[cn1,n22(ξ3)]=o(1n16n26).

Take the hyperbolic cross truncations of the expansion (111),

(117) TN(h)(ξ;t1,t2)=Pξ(t1,t2)+SN(φ1;t1)p(1−t1) +SN(φ4,t1)p(t1) +SN(ξ1(0,1)(t1,0))p(1−t2) +SN(ξ1(0,1)(t1,1))p(t2)+SN(h)(ξ3;t1,t2),

where each *S*_*N*_(*φ*_*i*_) is the first *N* terms partial sums of Fourier cosine series of *φ*_*i*_ and *S*_*N*_^(*h*)^(*ξ*_3_) is the hyperbolic cross truncation of Fourier cosine series of bivariate stochastic process of *ξ*_3_ which is stated in (21). The hyperbolic cross truncation *T*_*N*_^(*h*)^(*ξ*) is a combination of stochastic polynomials and cosine polynomials. By (111) and (117),

(118) TN(h)(ξ;t1,t2)−ξ(t1,t2)=(SN(φ1;t2)−φ1(t2))p(t1) +(SN(φ2;t2)−φ2(t2))p(t1) +(SN(φ3;t1)−φ3(t1))p(t2) +(SN(φ4;t1)−φ4(t1))p(t2) +(SN(h)(ξ3;t1,t2)−ξ3(t1,t2)).

Again, by the formula (∑_*n*=1_^*k*^ *a*_*n*_)^2^ ≤ *k* ^2^∑_*n*=1_^*k*^ *a*_*n*_^2^, we get

(119) 125E[||TN(h)(ξ)−ξ||22] ≤(∑i=14E[||SN(φi)−φi||22])||p||22  +E[||SN(h)(ξ3)−ξ3||22].

By using Parseval identity and (114), we get

(120) E[||SN(φi)−φi||22]=12∑n=N∞E[cn2(φi)]=o(1N5)(i=1,2,3,4).

By Proposition 2, we have *ξ*_3_^(1,1)^(0,0) = *ξ*_3_^(1,1)^(0,1) = *ξ*_3_^(1,1)^(1,0) = *ξ*_3_^(1,1)^(1,1) = 0. Based on (114) and (116), the argument similar to Theorem 7 shows that

(121) $$\begin{array}{c} E\left[{||S_{N}^{\left(h\right)}(\xi_{3})-\xi_{3}||}_{2}^{2}\right]=o\left(\frac{\log N}{N^{5}}\right). \end{array}$$

From this and (119), we deduce that *E*[||*T*_*N*_^(*h*)^(*ξ*)−*ξ*||_2_^2^] = *o*((log⁡*N*)/*N* ^5^). Noticing that the number of Fourier cosine coefficients in *T*_*N*_(*t*_1_, *t*_2_) : *N*_*c*_ ~ *N*log⁡*N*, we get the following.

**Theorem 8 .**

Suppose that *ξ* is a stochastic process on [0,1]^2^ and *ξ* ^(*l*,*l*)^ ∈ *C*_*s*_([0,1]^2^) for some *l* ≥ 3. Then

(122) $$\begin{array}{c} E\left[{||T_{N}^{\left(h\right)}\left(\xi \right)-\xi ||}_{2}^{2}\right]=o\left(\frac{\log^{6}N_{c}}{N_{c}^{5}}\right), \end{array}$$

where *N*_*c*_ is the number of Fourier cosine coefficients in *T*_*N*_^(*h*)^(*ξ*).

Comparing Theorem 8 with (108) and (109), we obtain the following.

**Theorem 9 .**

Let *ξ* be a stochastic process on [0,1]^2^ and *ξ* ^(*l*,*l*)^ ∈ *C*_*s*_([0,1]^2^) for some *l* ≥ 3. Then

(123) E[||SN(r)(ξ)−ξ||22]~1Nc3/2,E[||SN(h)(ξ)−ξ||22]~log⁡4NcNc3,E[||TN(h)(ξ)−ξ||22]=o(log⁡6NcNc5),

where *S*_*N*_^(*r*)^(*ξ*) and *S*_*N*_^(*h*)^(*ξ*) are partial sums and hyperbolic cross truncations of Fourier cosine series of *ξ*, respectively, *T*_*N*_^(*h*)^(*ξ*) is the hyperbolic cross truncations of Fourier expansion of *ξ* with polynomial factors, and *N*_*c*_ is the number of Fourier cosine coefficients in each sum.

From this, we see that if we reconstruct *ξ* by *T*_*N*_^(*h*)^(*ξ*), we need the least Fourier cosine coefficients.
